# Integrative molecular characterization of pediatric spinal ependymoma: the UK Children’s Cancer and Leukaemia Group study

**DOI:** 10.1093/noajnl/vdab043

**Published:** 2021-03-08

**Authors:** Omar Ahmad, Rebecca Chapman, Lisa C Storer, Li Luo, Paul R Heath, Linda Resar, Kenneth J Cohen, Richard G Grundy, Anbarasu Lourdusamy

**Affiliations:** 1 Children’s Brain Tumour Research Centre, School of Medicine, University of Nottingham, Nottingham, UK; 2 The Johns Hopkins University School of Medicine, Baltimore, Maryland, USA; 3 Sheffield Institute for Translational Neuroscience, University of Sheffield, Sheffield, UK

**Keywords:** DNA methylation, ependymoma, myxopapillary, pediatric, spinal tumors

## Abstract

**Background:**

Pediatric spinal ependymomas (SP-EPNs) are rare primary central nervous system tumors with heterogeneous clinical course. Considering that ependymomas in children are biologically distinct from their adult counterparts, this study aimed to define the molecular landscape of SP-EPNs in children.

**Methods:**

In this retrospective study, we have collected tumor samples from 27 SP-EPN patients younger than 18 years and carried out the histological review, DNA methylation, and gene expression profiling.

**Results:**

Unsupervised analyses with methylation profiles revealed 2 subgroups where all grade I tumors (*n* = 11) were in Group 1, but the grade II/III tumors split into 2 groups (*n* = 7 in Group 1 and *n* = 9 in Group 2). The Heidelberg classifier assigned Group 1 tumors as spinal myxopapillary ependymomas (SP-MPEs), 5 Group 2 tumors as SP-EPNs, and failed to classify 4 Group 2 tumors. Copy numbers derived from DNA methylation arrays revealed subgroup-specific genetic alterations and showed that SP-EPN tumors lack *MYCN* amplification. Gene expression profiling revealed distinct transcriptomic signatures, including overexpression of genes involved in oxidative phosphorylation in SP-MPEs that were validated by Western blot analysis. We discovered widespread decreases in DNA methylation at enhancer regions that are associated with the expression of oncogenic signaling pathways in SP-MPEs. Furthermore, transcription factor motifs for master regulators, including *HNF1B*, *PAX3*, and *ZIC3*, were significantly overrepresented in probes specific to distal regulatory regions in SP-MPEs.

**Conclusion:**

Our findings show substantial heterogeneity in pediatric SP-EPN and uncover novel enhancers and transcriptional pathways specific to the SP-MPE subgroup, providing a foundation for future therapeutic strategies.

**Key Points**

- More than half of grade II/III spinal tumors in our cohort are molecularly classified as SP-MPEs.
- *MYCN* amplification is absent in all except one grade II/III spinal tumor.
- A widespread decrease in methylation at enhancer regions distinguishes SP-MPEs from SP-EPNs.

**Importance of the Study**

Our study, for the first time, describes the molecular characterization of rare spinal tumors in children. The DNA methylation array analysis segregates 27 pediatric SP-EPNs into 2 robust primary molecular subgroups. All grade I, myxopapillary tumors are grouped whereas grade II/III spinal tumors are distributed into 2 molecular subgroups. The Heidelberg methylation classifier assigns tumors to previously defined subgroups: SP-MPEs, SP-EPNs, and fails to classify 4 tumors as spinal ependymal tumors. None of the classified tumors has MYCN amplification in our cohort. Tumor samples in the SP-MPE subgroup show high expression of genes associated with oxidative phosphorylation and a widespread decrease in DNA methylation at enhancer regions compared to the SP-EPN subgroup. These findings enhance our understanding of the molecular landscape of the pediatric SP-EPN and point to subgroup-specific features for evaluation in future clinical trials.

Ependymoma is a malignant central nervous system tumor that accounts for only 8%–10% of all CNS tumors in children,^1,2^ although it is associated with adverse outcomes in younger patients compared to adults. Importantly, the molecular basis for these differences has remained elusive. Ependymoma occurs throughout the neuraxis—posterior fossa, supratentorial, and spinal cord, presenting in both adults and children. The majority of childhood ependymomas (90%) occur intracranially, whereas most tumors in adults arise in the spinal cord.^3^ Spinal ependymoma presents as a centrally located mass in the spinal cord that grows slowly and has demarcated borders. Pathologically, spinal ependymomas are classified by the WHO as grade I, grade II, or grade III. Recently, 9 molecular subgroups, with 3 subgroups in each of the anatomical regions (posterior fossa, supratentorial, spinal), were described using DNA methylation profiling.^4^ The 3 subgroups specific to spinal ependymoma included subependymoma (SP-SE), myxopapillary ependymoma (SP-MPE), and ependymoma (SP-EPN) and showed concordance with the histopathological subtypes. Amplification of the oncogene *MYCN* is observed in small series of SP-EPNs in 3 recent studies, 4 grade III tumors,^5^ 3 grade II and 10 grade III tumors,^6^ and 8 grade III tumors,^7^ and proposed as an additional subgroup of spinal ependymoma (SP-EPN-MYCN). Although recurrent oncogenic drivers have not been identified, SP-SE harbored 6q deletions, while SP-MPE and SP-EPN demonstrated chromosomal instability. Moreover, most SP-EPN tumors had a loss of the 22q locus, which harbors the tumor-suppressor gene, neurofibromin (NF2). Importantly, there is a paucity of studies focused on pediatric SP-EPNs since these tumors account for less than 10% of ependymomas in children.^4,8^ For example, the DNA-based classification study with 500 tumor samples had only 2 SP-EPNs from pediatric patients.^4^

Given the anatomic location of SP-EPNs in children, therapeutic options are limited and associated with significant complications. Most pediatric SP-EPNs, especially grade I, occur in the distal regions of the spinal cord, designated the filum terminale or conus regions.^9^ Currently, the only effective treatment is maximal safe surgical resection. Unfortunately, complete resection can only be achieved in patients with focal disease. Radiotherapy is generally recommended following incomplete excision, particularly for the grade II and III tumors. However, there is a lack of evidence regarding the utility of postoperative radiotherapy, with no consistent improvement in outcomes.^10,11^ Moreover, children with SP-EPN are more likely to experience a more aggressive disease, with higher rates of local failure following treatment and more frequent metastasis throughout CNS.^12^

Because these tumors are uncommon, the molecular underpinnings of pediatric SP-EPNs remain unknown. We hypothesized that molecular heterogeneity exists within pediatric SP-EPNs and, further, that understanding the heterogeneity will shed light on tumor development, personalized therapies, or potential therapeutic targets. To test these hypotheses, we performed multi-platform molecular profiling of SP-EPNs samples from 27 pediatric patients. Our findings reveal 2 distinct groups of pediatric SP-EPNs that correspond with recently defined DNA methylation-based molecular subgroups. We discovered that these molecular subgroups show distinct chromosomal copy number, transcriptional networks, and epigenetic alterations. Finally, we discovered novel enhancer regions from genome-wide methylation alterations in pediatric SP-EPNs.

## Materials and Methods

### Study Cohort

All tissue samples were radiotherapy and chemotherapy-naive and prospectively collected either (1) at the local tissue bank at Nottingham University Hospital (local ethics reference 11/EM/0076) or (2) as part of the Ependymoma clinical trials, coordinated by the University of Nottingham, United Kingdom (SIOP 1992,^13^ 1999^14^). The histopathology of all tumor samples was locally reviewed in each center and confirmed by central pathology review in Nottingham. Clinical information was obtained via the Children’s Cancer and Leukaemia Group, United Kingdom. The study was conducted following the Declaration of Helsinki. Tissue samples were obtained during the surgical resection of the tumor. All frozen samples were snap-frozen and stored at −80°C. Formalin-fixed paraffin-embedded (FFPE) tissue was collected as scrolls or unstained slides. A total of 27 patients with SP-EPN were included in the study.

### RNA and DNA Extraction

For frozen tissue tumor samples, RNA was extracted using miRNeasy Micro Kit (QIAGEN) and DNA was extracted using QIAamp DNA Mini Kit (QIAGEN), according to manufacturer’s guidelines. For FFPE tumor samples, RNA and DNA were extracted simultaneously using AllPrep DNA/RNA Mini Kit (QIAGEN) according to the manufacturer’s guidelines. Following RNA extraction, DNase I digestion step was carried out to remove DNA fragments. RNA quality control check was performed before microarray profiling. Similarly, DNA extraction was followed by bisulfite treatment before DNA methylation (DNA methylation: Zymo, product code D5001; DNA cleanup & concentrator-5: Zymo, D4013).

### Genome-Wide DNA Methylation Profiling

Samples were analyzed on the human Infinium MethylationEPIC BeadChips or the Illumina Infinium HumanMethylation450K at the UCL Genomics according to the manufacturer’s instructions. All analyses were performed in the R Statistical Environment (v3.6.0). Raw genome-wide methylation data (IDAT files) generated from both frozen and FFPE derived tissue were preprocessed using the minfi Bioconductor package version 1.30.0.^15^ Illumina 450K and EPIC BeadChip arrays were combined by selecting common probes that are present on both arrays. The Illumina preprocessing method that includes background subtraction and control normalization was selected to generate methylated values. To account for possible batch effects due to divergent protocols for fresh frozen and FFPE material, and type of array (450K or EPIC), a batch adjustment was performed using a linear model.^16^ The residuals from the linear model were back-transformed to the intensity scale, and methylation beta values were calculated as described in Illumina’s protocols. Subsequently, probes were removed according to the following filtering criteria: (1) mapping to the X and Y chromosomes, (2) containing a single-nucleotide polymorphism (dbSNP137 Common) at the targeted CpG site or the single-base extension site, (3) nonspecific probes, and (4) not unique to the human reference genome (hg19) allowing for one mismatch. In total, 401 060 probes were retained for downstream analysis. The SP-EPN molecular subgroup status was determined by the Heidelberg brain tumor classifier version 2.0 (https://www.molecularneuropathology.org/mnp).

### Gene Expression Profiling

RNA was hybridized to Human Affymetrix Clariom TM D arrays according to the manufacturer’s instructions. These arrays have more than 6.5 million probes interrogating both protein-coding and long noncoding genes. Array data were preprocessed with the Robust Multi-array Average method using the Affymetrix Power Tool (APT v2.10.2.2) and annotated with NetAffx annotation Release 36.^17^ The log_2_ transformed expression values for protein-coding genes were used for the subsequent analyses. Expression values were mapped from probe set to a unique gene and the probe set with the highest variance in expression was selected when multiple probe sets were mapped to the same gene. The final filtering step left a total of 25 272 genes.

### DNA Methylation-Based Clustering and Copy Number Alteration Analyses

Consensus clustering was performed to detect robust sample groups using the R Bioconductor package, ConsensusClusterPlus. The top 5000 CpG probes exhibiting the greatest median absolute deviation were used for the consensus clustering analysis. Specifically, 80% of the original 27 samples were randomly subsampled without replacement and partitioned into 2–10 major clusters using the hierarchical clustering (HCL) algorithm, which was repeated 1000 times. The number of clustering was determined by 3 factors: the average pairwise consensus matrix within consensus clusters, the delta plot of the relative change in the area under the cumulative distribution function curve, and the average silhouette distance for consensus clusters. Silhouette analysis was used to evaluate sample membership following consensus HCL, and SigClust was used to determine the statistical significance of subgroups. Also, a non-negative matrix factorization (NMF) clustering algorithm was used for the unsupervised subgroup discovery using the 5000 most variable CpG probes. NMF (R package: NMF version 0.21) was performed for 1000 resampling iterations using the default parameters. A comparison was made between consensus HCL and NMF using a Rand Index and assessed statistically by permutation of sample labels and repetition of the Rand Index calculation to generate a null distribution.

Copy number segmentation was performed from 450K and EPIC genome-wide methylation arrays using the conumee package version 1.17.0. Segment files were generated for each subgroup.

### Gene Set Enrichment Analysis of Gene Expression Data

Gene set enrichment analysis (GSEA) was performed using the javaGSEA application version 4.0.2. The gene list output from the differential expression analysis with an eBayes adjusted moderated t-statistic linear regression model was ranked by calculating a rank score of each gene as −log_10_ (*P* value) × sign (FC), in which FC is the fold change (expressed as log_2_ [expression in MPE/expression in SP]) and the sign depends on whether the gene is upregulated or downregulated.^16^ A pre-ranked GSEA analysis was performed using 1000 permutations. The gene sets from MSigDB collections, particularly C2: BioCarta and Kyoto Encyclopedia of Genes and Genomes pathway databases, and C5: Gene Ontology database were used for the GSEA analysis.

### Integrative DNA Methylation and Gene Expression Analysis

We used profiles from 27 pediatric SP-EPN tumor samples with both methylation and expression data sets. Using the Enhancer linking by methylation/expression relationships (ELMER) R package (version 2.6.1) in supervised mode, the methylation and expression data were integrated to investigate gene regulatory networks and identify related master regulator transcription factors.^18,19^ In short, for this analysis, the processed methylation data *β* values, as described above, were used, combined with processed microarray expression data. Only distal probes were selected, defined as probes at least ±2 kb away from a transcription start site (TSS). The moderated t-statistic based on the empirical Bayesian method (R package: Limma) was used to determine differentially methylated distal probes between 2 molecular subgroups.^16^ For these altered probes, the 20 closest genes (10 upstream and 10 downstream) were then identified and the association between probe methylation and gene expression was tested with a non-parametric Mann–Whitney *U* test, using the false discovery rate (FDR) for multiple testing correction. Significant probe–gene pairs were defined as pairs with an FDR less than 0.05. Next, a transcription factor binding motif enrichment analysis was performed on the regions (±250 bp) around the probes of significant hyper- and hypomethylated probe–gene pairs using Fisher’s exact test. A motif was considered enriched if the odds ratio was more than 1.1, it occurred more than 10 times in the probe set regions, and the FDR was less than 0.05. Finally, potential upstream master regulator transcription factors were determined by comparing the average methylation value of all probes associated with a motif with the expression values of genes annotated as a transcription factor with a Mann–Whitney *U* test. For this test, we assumed that lower transcription factor expression corresponds to increased methylation levels. After ranking by *P* value, the top 5% of transcription factors were considered master regulators.

### Western Blots

Normal human fetal spine protein lysate was purchased from the GeneTex International Corporation (product code GTX24529). Total protein was extracted from using the Qproteome FFPE Tissue Kit (QIAGEN) following the manufacturer’s guidelines. Extracted proteins were quantified using Bradford’s assay. For Western blot analysis, the total protein (30 µg) was loaded into 12% SDS-PAGE gels. Proteins were then transferred into polyvinylidene fluoride (PVDF) membranes using a wet transfer tool (Bio-Rad). Membranes were first blocked with 5% milk before probing with Total OXPHOS human WB antibody cocktail (Abcam, ab110411) at 4°C overnight. The next day, the membranes were washed with tris-buffered saline and Tween 20 (TBST) before probing with the secondary antibody (anti-mouse or anti-rabbit). Proteins were visualized via chemiluminescence (Signal Fire Elite ECL reagent, product number 12757P). Housekeeping protein GAPDH was used as an internal loading control (Cell Signalling, product code 97166). The Images were scanned using (FujiFilm 3000) and the resulting data were analyzed (Image J software).

## Results

Our SP-EPN cohort included 27 patients, all of whom were children younger than 18 years (median age 12.3 years; range 4.3–16.8 years). Of these, 17 were males and 10 were females. Histologic subtypes included tumors classified as WHO grade I (*n* = 11), WHO grade II (*n* = 14), and WHO grade III (*n* = 2; Table 1).

**Table 1.** Patient Characteristic of Pediatric Spinal Ependymoma Cohort

Patient Characteristics	*N* (%)
Age, years	
Median	12.3
Range	4.3–16.8
Sex	
Male	17 (63)
Female	10 (37)
Histologic diagnosis	
Myxopapillary ependymoma	11 (41)
Ependymoma	14 (52)
Anaplastic ependymoma	2 (7)
WHO tumor grade	
I	11 (41)
II	14 (52)
III	2 (7)
Initial tumor resection	
Partial	8 (30)
Complete	17 (63)
Missing	2 (7)
Follow-up	
Median follow-up (years)	3.6
Status	
Alive	24 (89)
Dead	2 (7)
Missing	1 (4)

### DNA Methylation Profiling Reveals 2 Molecular Subgroups of Pediatric S-EPN

**Figure 1.**
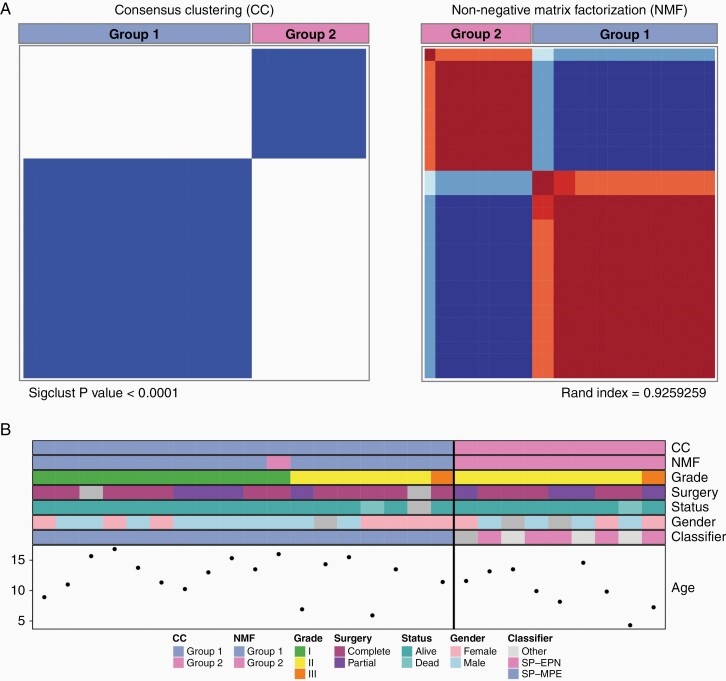
Pediatric spinal ependymomas comprise 2 subgroups. (A) Unsupervised consensus clustering (CC) and non-negative matrix factorization (NMF) heatmaps displaying the 2 robust subgroups defined by DNA methylation profiles from 27 pediatric pediatric spinal ependymomas using the top 5000 most variably methylated CpG probes across all tumors. Significance of clustering in CC was measured using SigClust. Sample overlap between 2 methods, CC and NMF, was measured using a Rand index. (B) DNA methylation-based subgroup assignment by 2 unsupervised methods (CC and NMF), clinical features, sample classification by the Heidelberg DNA methylation classifier (Heidelberg), and age at diagnosis (in years) are shown in colored tracks. Based on the assignments of the Heidelberg classifier, Group 1 was named as SP-MPE and Group 2 as SP-EPN.

To identify and validate molecular subgroups in our 27 pediatric S-EPNs, we analyzed DNA methylation array data from 27 S-EPN tumor samples by using 2 distinct unsupervised clustering algorithms. First, the consensus clustering with HCL analysis revealed 2 subgroups in S-EPNs, and the same groups were identified using the NMF method (Figure 1A and Supplementary Figure S1). Group 1 (*n* = 18) consisted of 11 WHO grade I, 6 WHO grade II, and 1 WHO grade III tumors whereas Group 2 consisted of both WHO grade II (*n* = 8) and III (*n* = 1) tumors. The Heidelberg brain tumor classifier on DNA methylation array data assigned all Group 1 tumors to “ependymoma, myxopapillary” and 5 Group 2 tumors to “ependymoma, spinal” (Figure 1B). The other 3 tumors in Group 2 were classified as “CNS high-grade neuroepithelial tumor with MN1 alteration,” “Plexus tumor,” and “control tissue, inflammatory tumor microenvironment,” and one with no matching methylation class (referred to as “Other” in Figure 1B). Based on these results, we annotated Group 1 as SP-MPE and Group 2 as SP-EPN (Figure 1B). To corroborate the clinical relevance of these subgroups, we analyzed 21 patients with known clinical outcome. We found that the SP-EPN subgroup was associated with unfavorable overall survival (OS) compared with SP-MPE although the difference in OS was not statistically significant (log-rank test, *P* value = .38; Supplementary Figure S2). The median OS for the SP-EPN subgroup was 5.33 years while the median for the favorable SP-MPE was not reached.

We further investigated WHO grade II and grade III S-EPNs of our cohort in more detail as these tumors were distributed to both molecular subgroups (Figure 1B and Supplementary Figure S3). One patient with WHO grade III in SP-EPN was a female (7.3 years old) who is free from relapse after the partial resection. In contrast, the WHO grade III female patient in SP-MPE was of 11.42 years of age with recurrence (time to recurrence: 2 years) after the complete resection surgery (Figure 1B and Supplementary Table S1). A comparison of clinical features revealed that patients with WHO grade II ependymoma in SP-EPN (*n* = 4) were younger than patients in SP-MPE (*n* = 6) subgroup (median 9.9 years vs 13.5 years). None of the patients with WHO grade II ependymoma in the SP-EPN subgroup relapsed or died from their disease, but one patient in SP-MPE did (Figure 1B) relapse and died. For the SP-MPE subgroup, 4 out of 5 WHO grade II ependymoma patients had a complete resection and showed a trend for better OS than all WHO grade II ependymoma patients in the SP-EPN subgroup (mean OS of 8.42 years vs 1.81 years).

### Pediatric S-EPN Subgroup-Specific Chromosomal Aberrations

**Figure 2.**
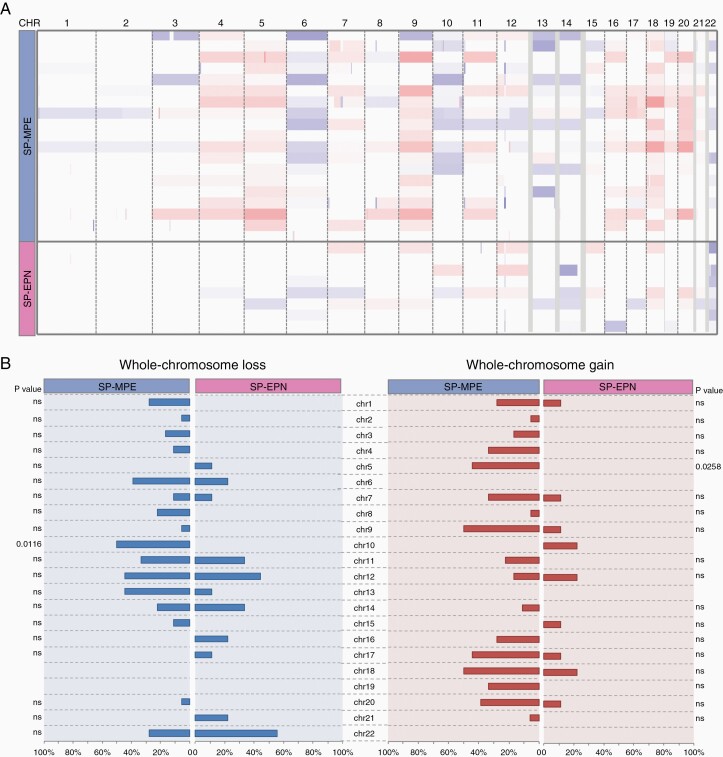
Copy number variations across 2 molecular subgroups of pediatric spinal ependymal tumors. (A) Genome-wide view of copy number alterations in 27 pediatric spinal ependymomas estimated from DNA methylation arrays subdivided by 2 subgroups: SP-MPE and SP-EPN. The heatmap displays gains (red) and losses (blue) of each chromosome (column) across each sample (row) subdivided by SP-MPE and SP-EPN. (B) Summary of chromosomal imbalances showing distinct alterations within 2 molecular subgroups of ependymal tumors. Copy number plots were generated based on DNA methylation, color code for losses (blue) and gains (red) of chromosomal profiles. Results were plotted as frequencies at which these aberrations occurred within each molecular subgroup; significance is illustrated by *P* values, which indicate distinct distributions of alterations across the molecular subgroups (chi-square test).

To identify genetic alterations that correlate with subgroups, we analyzed DNA copy number profiles using the combined intensity values of the methylated and unmethylated probes. Consistent with the previous genomic characterizations of ependymoma, all samples in SP-MPE and SP-EPN groups displayed aneuploidy (Figure 2A). The SP-MPE subgroup was characterized by a gain of chr5 and loss of chr10 (Figure 2B). Interestingly, the loss of chr22 was found in all 5 cases (4 WHO grade II and 1 WHO grade III EPN) in SP-EPN that were predicted as SP-EPN by the Heidelberg classifier but found in only one WHO grade II ependymoma (1 of 6 WHO grade II and 1 WHO grade III) of the SP-MPE subgroup. To further investigate pediatric spinal subgroups, we performed copy number analysis using GISTIC 2.0 that revealed a much lower number of focal copy number alterations. The only focal and recurrent amplification was observed in SP-MPE (4/18; 22%) encompassing HOXB cluster genes (17q21.32) that include 9 consecutive HOXB genes (*HOXB1*-B9) and 2 micro-RNAs (*miR-10a* and *miR-196a-1*; Supplementary Table S2). Less frequent recurrent deletions in and around *ALG10* (12q12; 3/18), *TP73* (1p36.32; 2/18) and significant non-recurrent deletions in and around *miR-202* (10q26.3), *POTEA* (8p11.1), *IDI1* (10p15.3), and *miR-483* (11p15.5) were found in SP-MPE (Supplementary Table S3). In contrast, the SP-EPN subgroup had no significant recurrent copy number alterations except the non-recurrent deletion around *ALG10* (12q12; 1/9). SP-EPN with *MYCN* amplification has been recently proposed as a new ependymoma subgroup. We observed *MYCN* amplification in one tumor, which was predicted to be a “Plexus Tumor” by the Heidelberg brain tumor classifier (Supplementary Table S1). Interestingly, this tumor sample was also classified as *MGMT* methylated.

### Pediatric SP-MPE Tumors Exhibit Increased Expression of Mitochondrial Metabolism Pathways

**Figure 3.**
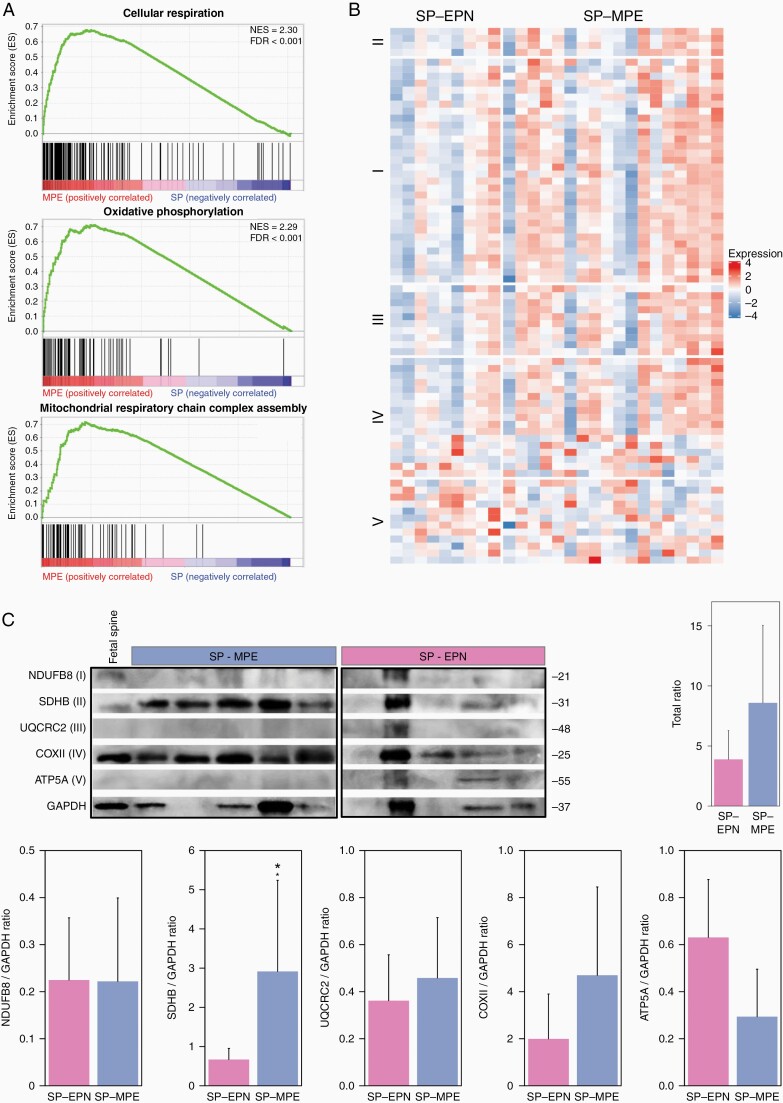
Mitochondrial metabolism pathways define the SP-MPE subgroup. (A) Gene set enrichment analysis plot for the top 3 significantly enriched signaling pathways: cellular respiration, OXPHOS pathway, and mitochondrial respiratory chain complex assembly identified on comparing SP-MPE against SP-EPN using gene expression profiles from 27 pediatric ependymomas. (B) Heatmap showing expression of genes that are associated with 5 different oxidative phosphorylation complexes. A row represents a gene and the column indicates the sample. The normalized expression value of each gene is indicated by color intensity, with red/blue representing high/low expression. (C) Western blot showing 5 OXPHOS proteins (ATP5A, COXII, NDUFB8, SDHB, and UQCRC2) in SP-MPE (*n* = 5) and SP-EPN (*n* = 5). I, II, III, IV, and V indicate OXPHOS complexes. GAPDH is internal control. Mean of 5 OXPHOS protein levels quantified from WB and normalized to GAPDH are shown as well as for each complex separately. *P* value from Mann–Whitney test. ATP5A, ATP synthase subunit alpha 5; COXII, cytochrome oxidase subunit II; NDUFB8, NADH: ubiquinone oxidoreductase subunit B8; SDHB, succinate dehydrogenase [ubiquinone] iron-sulfur subunit B; UQCRC2, ubiquinol-cytochrome C reductase core protein 2. *N* = 5 for both SP-MPE and SP-EPN.

To delineate characteristic biological processes and signaling pathways for each of these pediatric SP-EPN subgroups, we performed GSEA with the ranked gene list. We found that pathways involved in mitochondrial function (cellular respiration, oxidative phosphorylation, aerobic respiration, and mitochondrial translation) and structure (mitochondrial respiratory chain complex assembly, and electron transport chain), and cellular energy metabolism (cellular aldehyde metabolic process, nucleoside triphosphate metabolic process, and fatty acid beta-oxidation) defined SP-MPE (Figure 3A and Supplementary Table S4). There was no significant gene set enriched in SP-EPN at FDR less than 0.05; however, potassium ion transport was detected as the top gene set with FDR less than 0.17 (Supplementary Table S5). The majority of oxidative phosphorylation (OXPHOS) metabolic pathway genes across 5 complexes showed high expression in SP-MPE when compared to SP-EPN (Figure 3B). To validate the OXPHOS signature observed at the transcriptional level in SP-MPE, we performed Western blot analysis of OXPHOS complexes. We examined 5 OXPHOS proteins: NADH: ubiquinone oxidoreductase subunit B8 (*NDUFB8*); succinate dehydrogenase complex iron-sulfur subunit B (*SDHB*); ubiquinol-cytochrome c reductase core protein II (*UQCR2*); mitochondrially encoded cytochrome c oxidase II (*COXII*); ATP synthase, H^+^ transporting, mitochondrial F1 complex, alpha subunit (*ATP5A*) in pediatric S-EPN samples. We found that the total protein levels of 5 OXPHOS complexes were high in SP-MPE (8.6 ± 3.3) compared with SP-EPN (3.9 ± 1.2) and contributed by complexes II, III, and IV. Interestingly, the levels of complex V were reduced in SP-MPE and there were no differences in complex I across 2 subgroups (Figure 3C).

We next aimed at evaluating the previously identified subgroup-specific transcriptional signatures^4^ in our pediatric cohort of 27 SP-EPN patients. We observed consistent expression patterns of SP-MPE and SP-EPN subgroup-specific signature genes in pediatric SP-EPN (Supplementary Figure S4). More than half of the SP-EPN signature genes identified in our dataset, including *HOXA13*, *NEFL*, *HNF1B*, *KMO*, and *HOXB13*, showed increased expression in SP-MPE (FDR < 0.05, Wilcoxon’s rank-sum test), whereas 3 genes, *RAB3C*, *VEPH1*, and *WT1*, among the 18 SP-EPN signature genes showed high expression levels in the SP-EPN subgroup. While we observed gene signatures reflecting the underlying transcriptional profiles of pediatric SP-EPN subgroups, the power of this analysis was limited due to the small sample size.

### DNA Methylation Alterations Are Enriched for Probes at Enhancers and Developmental Transcription Factors Binding Sites

**Figure 4.**
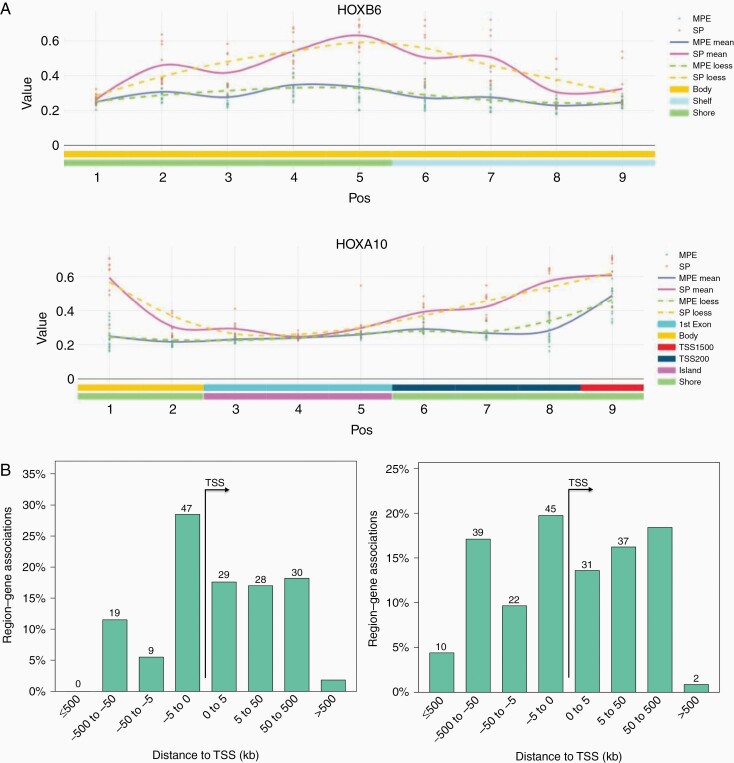

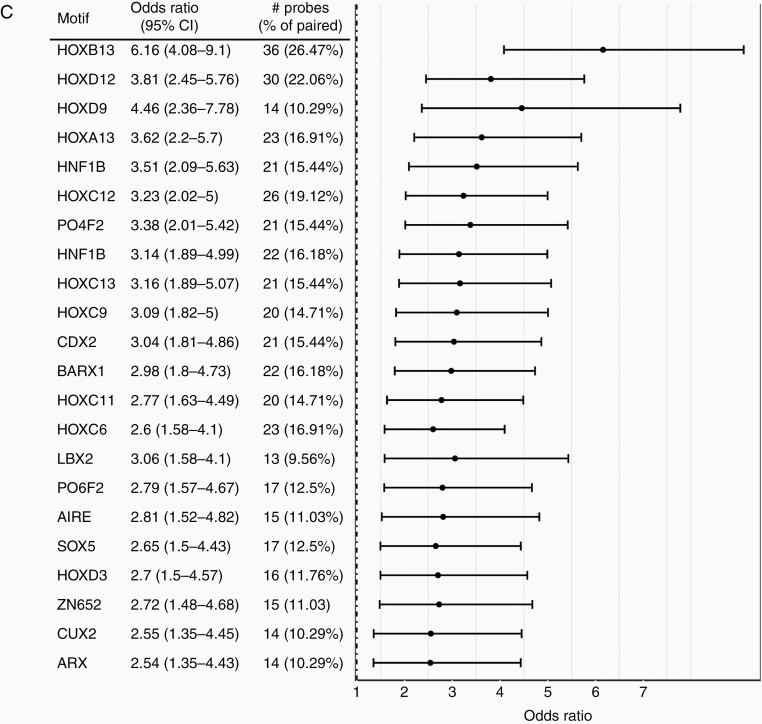
SP-MPE exhibits DNA methylation profiles distinct from SP-EPN. (A) Representative plot of the differentially methylated region (DMR) in SP-MPE compared to SP-EPN tumors for HOX genes (HOXB6 and HOXA10). The points represent DNA methylation levels (*β* value) for each tumor sample and smoothed lines indicate the mean *β* values at the CpG probe for SP-MPE and SP-EPN. (B) Bar graphs displaying association of genomic regions to the transcription start site (TSS) of all DMRs with low (left) and high (right) methylation levels in SP-MPE when compared with SP-EPN tumors. The distance between CpG probe location and their putatively regulated genes is divided into 4 separate bins (x-axis) and the percentage of genes in each genomic region (y-axis) is shown. The absolute number is also listed on the top of each bar graph. (C) Motif enrichment plot shows the enrichment levels (odds ratio >2.5) for the most significant motifs based on ELMER supervised analysis. A complete list of significant motifs (odds ratio >1.1) is presented in Supplementary Table 10.

To characterize candidate biological processes altered by differential methylation between the subgroups, we identified differentially methylated regions (DMRs) and performed the genomic regions enrichment analysis. DMRs with high methylation levels in SP-MPE (57% of 247 DMRs at FDR < 0.05) showed an unexpected enrichment for genes encoding immune-related proteins, including MHC protein complex (FDR = 2.5 × 10^−03^, Binomial test; *HLA-DMB*, *HLA-DOA*, and *HLA-G*) and TAP complex (FDR = 2.14 × 10^−03^; *TAP2* and *TAPBP*; Supplementary Table S6). We also observed a significant enrichment of genes involved in protein tyrosine kinase collagen receptor activity (FDR = 4.21 × 10^−03^) and HMG box domain binding (FDR = 9.69 × 10^−03^). In contrast, DMRs with low methylation levels in SP-MPE were enriched for several developmental processes, including anterior/posterior pattern specification (FDR = 3.95 × 10^–15^), trophoblast giant cell differentiation (FDR = 2.23 × 10^−03^), and cell fate specification (FDR = 2.01 × 10^−02^; Supplementary Table S7). We also observed a significant enrichment of HOX genes (*HOXA9*, 11, 13; *HOXB2*, 4, 5, 6, 9, 13; *HOXC9*, 10, 11; *HOXD3* and *HOXD4*) associated with low methylated DMRs in SP-MPE (Figure 4A). Of note, *HOX* gene overexpression is associated with SP-EPN, thus validating our approach.

The significant DMRs consisted of on average 10 CpG probes that spanned over 5 kb from the TSS, indicating DNA methylation changes preferentially occurring in distal regions (Figure 4B), suggesting enhancers were affected by changes in methylation. We, therefore, sought to identify subgroup-specific DNA methylation changes in distal enhancer regions. To this end, we selected CpG probes that are greater than ±2 kb from a known TSS, resulting in a set of 147 195 distal probes. The majority of distal probes had low methylation (91% of 1536 probes) versus high methylation levels for SP-MPE compared to SP-EPN (|∆β| > 0.2 and FDR < 0.05; Supplementary Figure S5). To identify target genes regulated by the distal regulatory elements, we analyzed expression data for 10 genes upstream and 10 genes downstream from each significant distal probe. We identified a total of 247 distal probe–gene pairs with low methylation and increased expression levels and only 4 distal probe–gene pairs with high methylation and decreased expression in SP-MPE (Supplementary Figure S6). Notably, oncogenes exemplified by *CDK6*, *HOXD11*, *IDH2*, and *JAK2*, and genes involved in oxidative phosphorylation (*COX4I1*, *DLAT*, *NDUFA5*, and *NDUFA6*), were overexpressed in SP-MPE and demonstrated low methylation (Supplementary Table S8). In contrast, long intergenic non-protein-coding RNAs (*LINC01102* and *LINC01114*) and the gene encoding POU-domain containing proteins (*POU3F3* and *PANTR1*) which have decreased expression in SP-MPE demonstrated high methylation levels (Supplementary Table S9). We next sought to determine which upstream transcription factors (TFs) control the activity of these regulatory elements. The integrative analysis with ELMER identified 38 significantly enriched TF binding motifs within candidate regulatory regions associated with 247 SP-MPE’s distal probes (Supplementary Table S10). The most highly enriched motifs corresponded to several homeobox (HOX) family members, including *HOXB13*, *HOXD12*, *HOXD9*, *HOXA13*, and *HOXC13* (Figure 4C). Considerable evidence demonstrates that when a group of enhancers is co-ordinately altered in a specific sample subset, which is often the result of an altered upstream master regulator TF in the gene regulatory network. Taking into account these known biological phenomena, we performed an integrative analysis, thereby identifying a small set of potential candidate master TFs, including homeodomain-containing TFs (*BARX2* and *HNF1B*) and zinc finger TFs (*ZIC3* and *ZNF423*; Supplementary Table S10).

## Discussion

During the past decade, advancing technologies have provided an unprecedented view of the genomic landscape of CNS tumor types, including ependymoma. These studies showed that ependymoma is a molecularly heterogeneous group of tumors, which can be classified into 9 different subgroups based on major CNS compartments. One limitation of these studies, however, is that the cohorts were comprised predominantly of young adult or adult patients. Importantly, ependymoma occurs in both pediatric and adult patients, and the location and behavior of the tumors vary in an age-dependent fashion. To elucidate genomic features specific to pediatric SP-EPN, we selected a cohort of 27 pediatric patients and performed an integrative DNA methylation and transcriptomics study. We discovered that SP-EPN in children is stratified into 2 subgroups with distinct genomic and transcriptomic features. To our knowledge, the discovery of 2 molecular subgroups in this study represents the first effort to characterize SP-EPN in children. Notably, these primary subgroups emerge from unsupervised analysis and are well-supported by a recently proposed DNA methylation-based classifier for CNS tumors.

The majority of pediatric SP-EPNs in our cohort could be assigned to 1 of 2 recently defined DNA methylation-based ependymoma subgroups: SP-MPE or SP-EPN.^4^ All pediatric patients with histologically defined WHO grade I showed a perfect match with SP-MPE, whereas WHO grade II and grade III tumors were heterogeneous by DNA methylation profiling and distributed to both subgroups. The importance of this change in-group allocation from histology to the molecular group for a subset of pediatric tumors is unclear. In fact, the Consortium to Inform Molecular and Practical Approaches to CNS Tumor Taxonomy (cIMPACT-NOW) working group recommended designating myxopapillary ependymoma as WHO grade II in their most recent update 7.^20^ Nevertheless, pediatric patients with WHO grade II or aggressive WHO grade III tumors in the SP-MPE subgroup are slightly older and with longer survival compared to tumors of the same grades in SP-EPN. Interestingly, DNA methylation profiling of adult ependymomas had previously shown that a subset of spinal WHO grade II tumors could be classified as SP-MPE.^21^ Furthermore, a study with gene expression profiling of 35 SP-EPNs (~80% from adult patients) demonstrated that myxopapillary and WHO grade II SP-EPN are molecularly distinct entities and a subset of grade II tumors could either be clustered with the myxopapillary group or not be firmly assigned to any subgroup.^22^ Together, these findings indicate that WHO grade II SP-EPNs are heterogeneous and histopathological evaluation alone is not sufficient. Thus, DNA methylation profiling may help refine the classification of these tumors.

In our study, the complete or partial gain of chromosome 5 and loss of chromosome 10 were the most significant genomic alterations, occurring commonly in SP-MPE tumors, but not in SP-EPN. Also, a relatively low number of focal and recurrent alterations was detected in our cohort, which is consistent with previous genomic studies of ependymoma. Strikingly, the only recurrent, focal amplification on the chromosomal arm 17q involving *HOXB* cluster genes at 17q21 was found in SP-MPE tumors. Interestingly, *MYCN* amplification was detected in one grade II spinal tumor that was classified as a “Plexus tumor” by the Heidelberg brain tumor classifier. All other grade II/III spinal tumors in our cohort did not harbor *MYCN* amplification, the finding contrasts with recent studies,^6^ where an aggressive subgroup of SP-EPN characterized by *MYCN* amplification (SP-EPN-MYCN) was identified. The 3 SP-EPN-MYCN cohort from previously published studies consisted of a total of 25 patients with a median age of 32 years, suggesting that the extremely rare *MYCN* amplification is likely to be a specific characteristic of SP-EPN from adult patients with highly aggressive disease.^6^ Less frequent or non-recurrent focal and statistically significant deletions found in SP-MPE included genes involved in the p53 pathway (*CD81*, *HRAS*, *CTSD*, *WRAP73*, and *PIDD1*). Although the loss of chromosome 22 was noted in all grade II tumors of SP-EPN, it was not frequently found in our grade II tumors assigned to the SP-MPE group. It remains to be seen whether the loss of chromosome 22 plays a role in grade II SP-EPNs in children.

In addition to genomic differences, pediatric spinal ependymal tumors showed subgroup-specific transcriptional differences. For instance, cellular and mitochondrial energy metabolism genes were most active in SP-MPE tumors, in line with previous data for the SP-MPEs.^22,23^ Notably, a number of genes related to the OXPHOS pathway were overexpressed in the SP-MPE subgroup compared to the SP-EPN subgroup. Our integrative analysis of DNA methylation and gene expression at enhancer regions also suggested the involvement of OXPHOS genes in SP-MPE tumors, providing evidence of the underlying biology and pathway activation in the SP-MPE subgroup. Previous pathological studies showed that myxopapillary ependymomas were frequently positive for *COX2*,^24,25^ a complex IV subunit of the mitochondrial respiratory chain, OXPHOS, which fits with our present results. A large body of evidence suggests that *COX2* is upregulated in various cancers and plays roles in promoting cell proliferation, neovascularization, growth, and metastasis of tumor cells.^26–28^ While this observation is interesting, definite conclusions regarding *COX2* detection and its role in pediatric SP-MPE tumors will have to await validation in larger patient cohorts. Besides OXPHOS pathway activation, our gene expression analyses furthermore showed high expression of previously identified SP-MPE subgroup-specific signature genes, including *HNF1B* and *HOXB13* that belong to the homeobox gene family. Several previous studies revealed that homeobox genes, especially *HOXB13*, were exclusively expressed in spinal myxopapillary,^29,30^ indicating *HOXB13* as a diagnostic marker to distinguish SP-MPE subgroup from other spinal subgroups. It is also worth noting that the homeobox family of genes play important roles in stem cell renewal, cell fate determination, and specify the patterning of body segments along the anterior–posterior axis, where *HOXB13* expression is limited to the lumbar/sacral regions of the spinal cord, the prominent locations for myxopapillary ependymomas.^23,29,31^ Furthermore, our study revealed enrichment for homeobox TF binding motifs in SP-MPE tumors with methylated DMR patterns at enhancer regions. These master regulatory TFs include *HNF1B*, a member of the hepatocyte nuclear factor family, which regulates the complex gene networks involved in lipid, carbohydrate, and protein metabolism and plays an important role in embryonic morphogenesis. Translational studies to validate these findings and provision of greater details in the various manifestations of associated processes may reveal new opportunities for targeted therapies for these rare SP-MPE tumors in children.

A major limitation of studying a rare cancer such as pediatric SP-EPN is the relatively low number of tumor samples with available tissue for a more comprehensive molecular profiling. Multivariable analysis with regard to survival and prognostic factors was not feasible due to small patient numbers. The validation in a larger and independent pediatric SP-EPN cohort is needed to further establish the difference between SP-MPE and SP-EPN including clinical prognosis.

In summary, we uncovered 2 molecular subgroups of pediatric SP-EPN that differ in their genomic and transcriptional characteristics. Specifically, we discovered that transcriptional networks associated with oxidative phosphorylation were enriched in SP-MPE. Furthermore, our results suggest that alterations in methylation patterns at key enhancers contribute to alterations in gene expression. Finally, we identified putative master regulatory TFs that may govern these changes in gene expression. This multifaceted analysis provides a valuable resource for assessing genomic alterations across the spectrum of pediatric spinal tumors. While further studies with whole-genome and transcriptome analysis in expanded cohorts are warranted, this study provides a strong basis for functional follow-up and investigation of potential therapeutic targets in young patients with SP-EPN.

## Supplementary Material

vdab043_suppl_Supplementary_Figures

vdab043_suppl_Supplementary_Tables

## Notes

**Authorship Statement.** Conception and design: R.G.G. and A.L.; development of methodology: O.A. and A.L.; acquisition of data: O.A., R.C., and P.H.; analysis and interpretation of data: O.A. and A.L.; writing, review, and/or revision of the manuscript: O.A., L.L., P.H., L.R., K.C., R.G.G., and A.L.; administrative, technical, or material support: R.C., L.S., and P.H.; study supervision: R.G.G. and A.L.
