# Supplementary material for: Integrative molecular characterization of pediatric spinal ependymoma: the UK Children’s Cancer and Leukaemia Group study
Source: Neurooncol Adv. 2021 Mar 8;3(1):vdab043. doi: 10.1093/noajnl/vdab043 (PMC8134525; doi:10.1093/noajnl/vdab043)
Supplement: vdab043_suppl_Supplementary_Figures [file vdab043_suppl_supplementary_figures.pdf]

Supplementary material

Integrative molecular characterization of pediatric spinal ependymoma: The UK Children's Cancer and Leukaemia Group study

Omar Ahmad[1], Rebecca Chapman[1], Lisa C. Storer[1], Li Luo[2], Paul R. Heath[3], Linda Resar[2], Kenneth J. Cohen[2], Richard G. Grundy[1], Anbarasu Lourdusamy[1]

Figure S1. Unsupervised analyses of 27 pediatric spinal ependymomas using DNA methylation profiles ………………………………………………………………………………3

Figure S2. Assignment of grade II and III pediatric spinal ependymomas using DNA methylation profiles………………………………………………………………………………5

Figure S3. Assignment of grade II and III pediatric spinal ependymomas using DNA methylation profiles………………………………………………………………………………5

Figure S4. Subgroup-associated gene signature ……….6

Figure S5. Differentially methylated distal probes between SP-MPE and SP-EPN subgroups of pediatric spinal ependymoma………………………………………………7

Figure S6. DNA methylation CpG probe-gene pairs correlation between methylation and gene expression………………………………………………………………………..8

Table S1. Clinical and histopathological data associated with all pediatric spinal ependymomas analysed in this study, along with molecular subgroup assignment

Table S2. Summary of focal amplifications detected in spinal ependymoma by GISTIC2

Table S3. Summary of focal deletions detected in spinal ependymoma by GISTIC2

Table S4. List of significant pathways enriched by Gene Set Enrichment Analysis in SP-MPE subgroup

Table S5. List of significant pathways enriched by Gene Set Enrichment Analysis in SP-EPN subgroup

Table S6. List of significant pathways enriched in differentially methylated regions (DMR) that have high methylation levels in SP-MPE

Table S7. List of significant pathways enriched in differentially methylated regions (DMR) that have low methylation levels in SP-MPE

Table S8. List of CpG probe - gene pairs with low methylation and high gene expression levels in SP-MPE

Table S9. List of CpG probe - gene pairs with high methylation and low gene expression levels in SP-MPE

Table S10. Summary of significant transcription factor (TF) with motifs enriched among the list of CpG probe - gene pairs with high methylation and low gene expression levels in SP-MPE

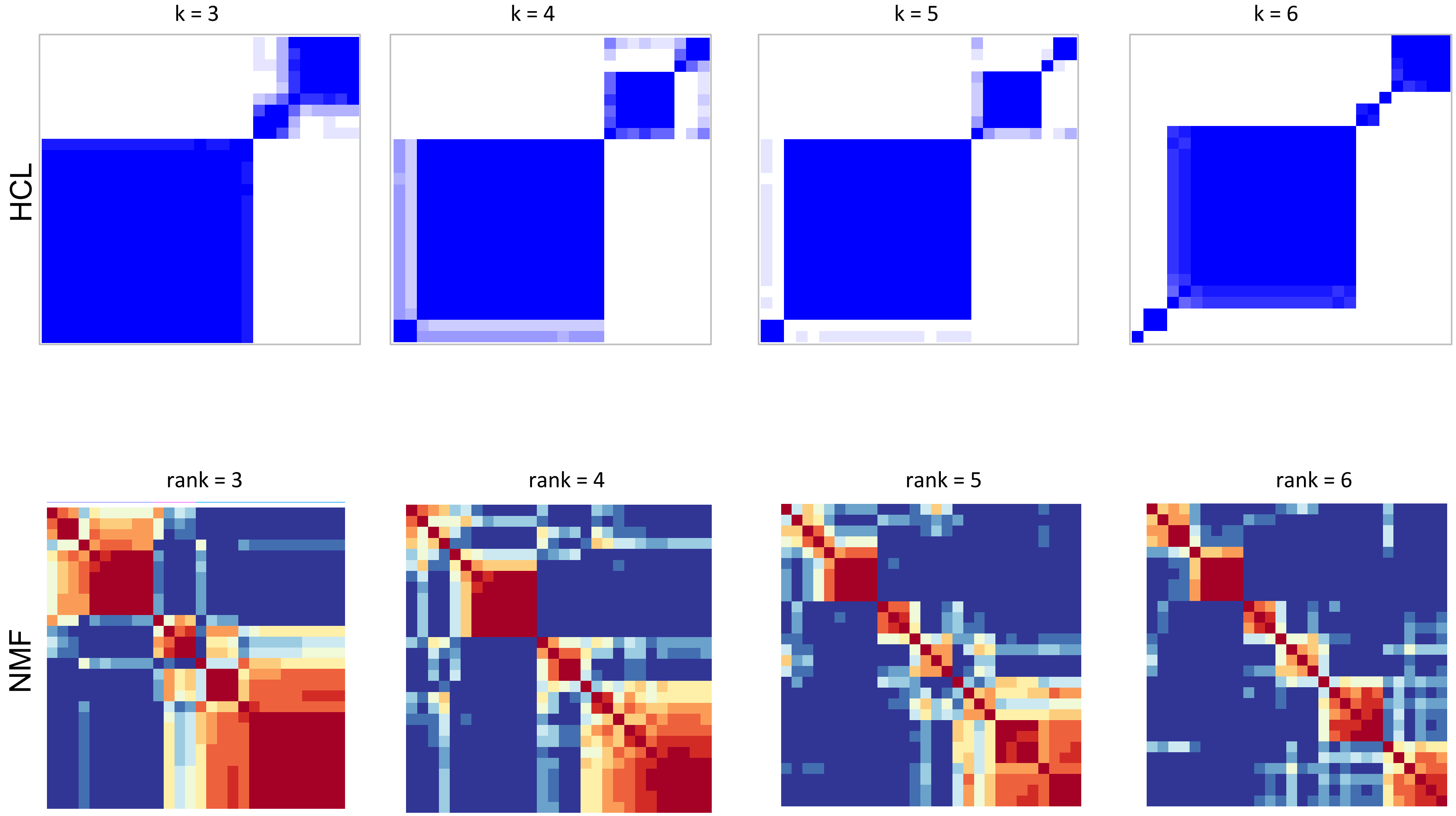

Supplementary figure S1. Unsupervised consensus hierarchical clustering (HCL) and non-negative matrix factorization (NMF) of 27 pediatric spinal ependymoma DNA methylation from a rank 2 to 6 classification.

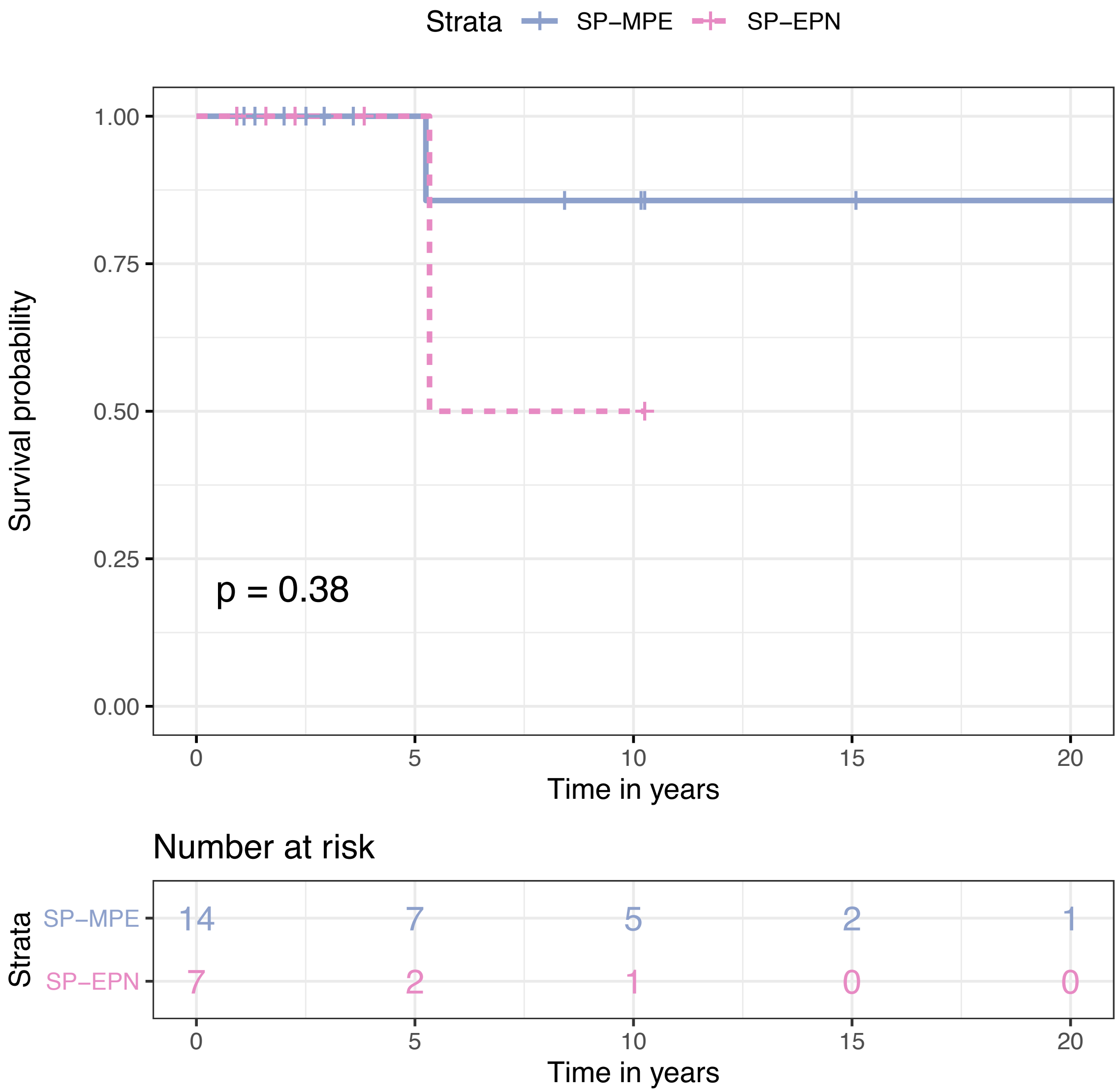

Supplementary figure S2. Kaplan-Meier curves of overall survival for our cohort when the patients are stratified by molecular subgroups, SP-MPE and SP-EPN. The median overall survival for the SP-EPN was 5.33 and was not reached for the SP-MPE. Significance levels were determined by log-rank test.

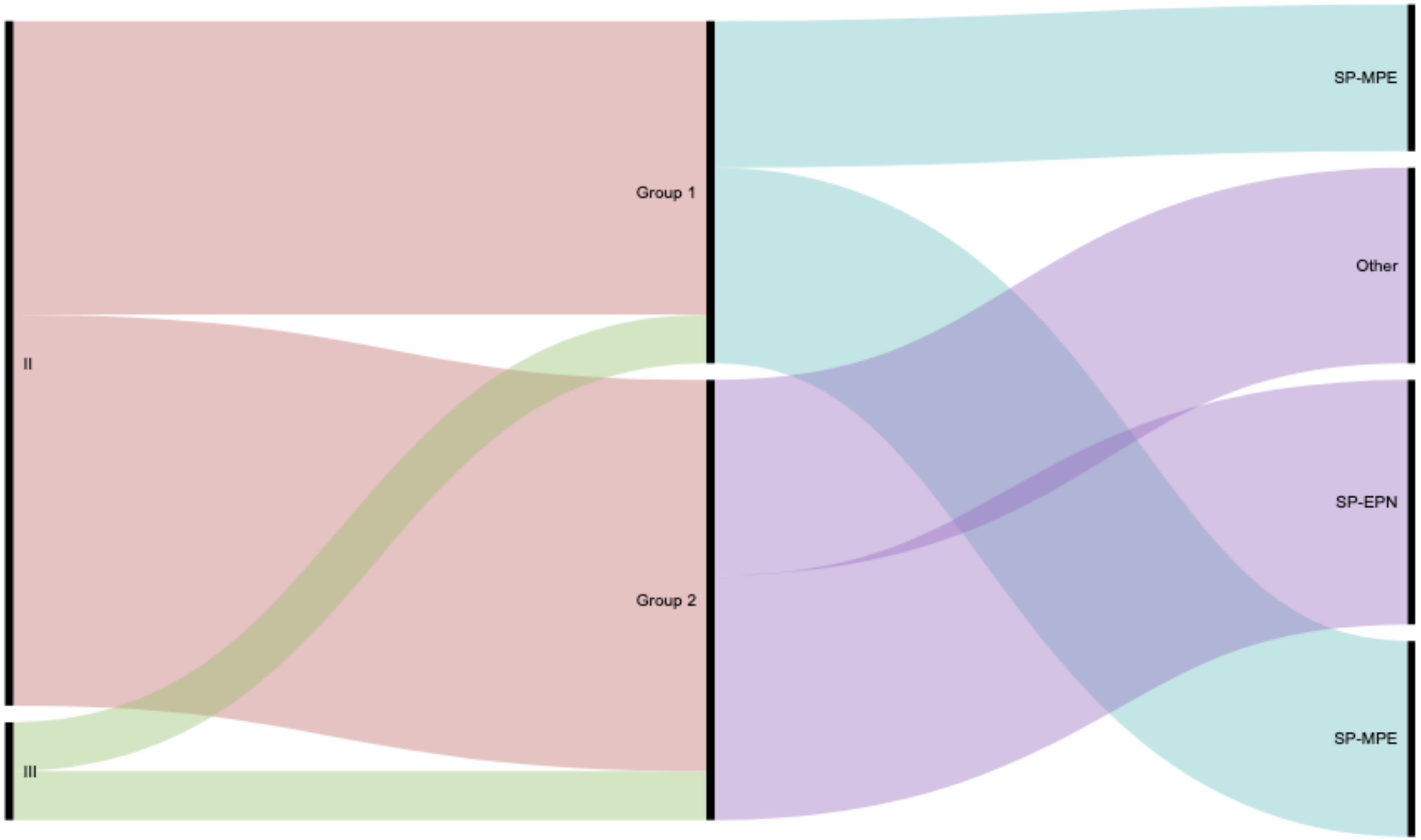

Supplementary figure S3. Assignment of grade II and III to molecular ependymoma subgroups based on DAN methylation profiles in our pediatric spinal ependymoma cohort.

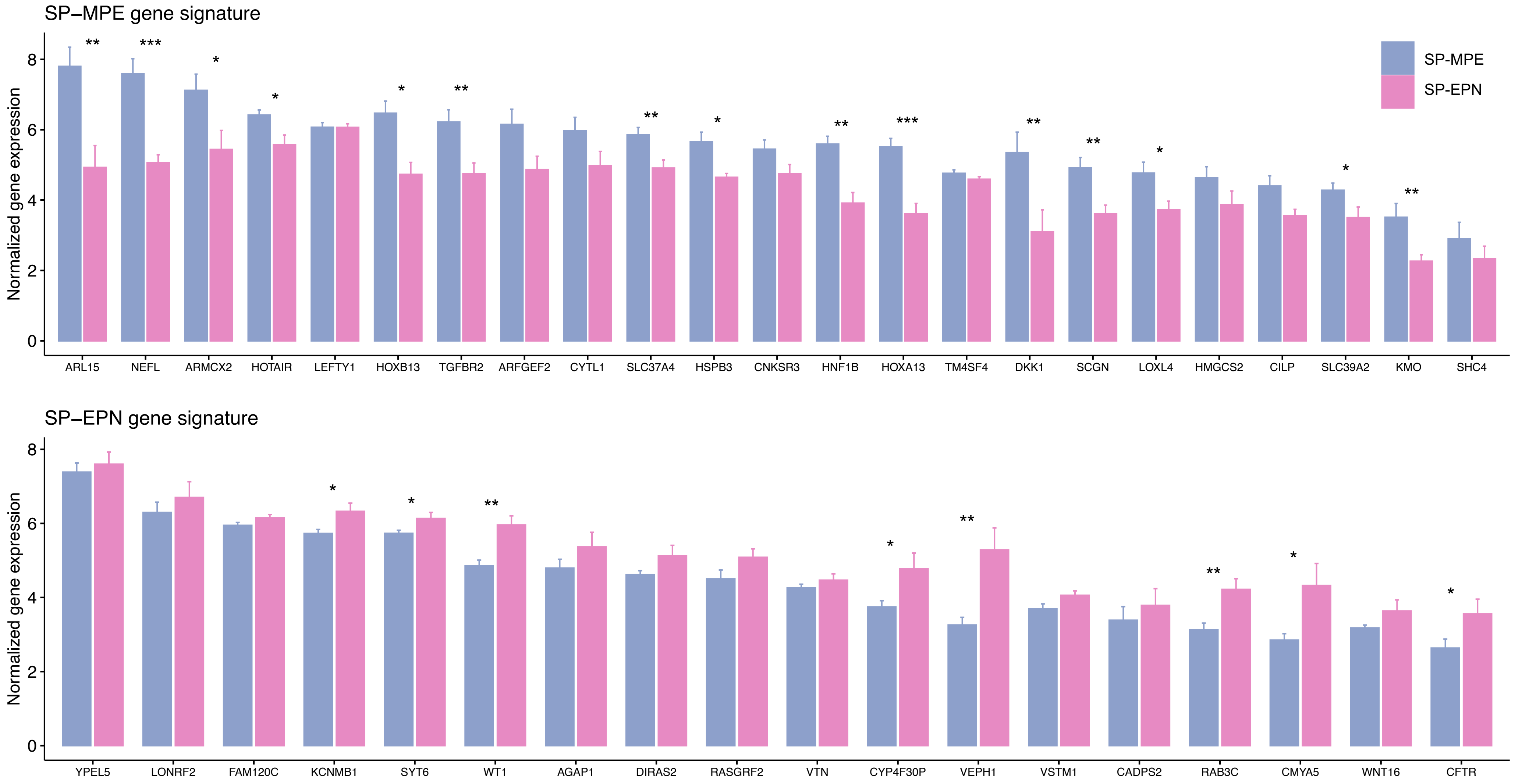

Supplementary figure S4. Subgroup-associated gene signature. Bar plot shows the expression levels of signature genes for SP-MPE (top) and SP-EPN subgroups (bottom). The subgroup-specific gene signatures were obtained from the previously published study by Pajtler et.al (2015). The Wilcoxon signed-rank test was used and p values (*: $p < 0.05$; **: $p < 0.01$; ***: $p < 0.001$) on the top indicate whether there was a significant difference in the distribution of expression values across the two subgroups, SP-MPE and SP-EPN. The p values for all SP-MPE signature genes remain significant after adjusting for multiple comparisons (false discovery rate, FDR) using the Benjamini-Hochberg adjustment whereas only three genes: *RAB3C*, *VEPH1* and *WT1* remain significant for the SP-EPN gene signature at the FDR < 0.05.

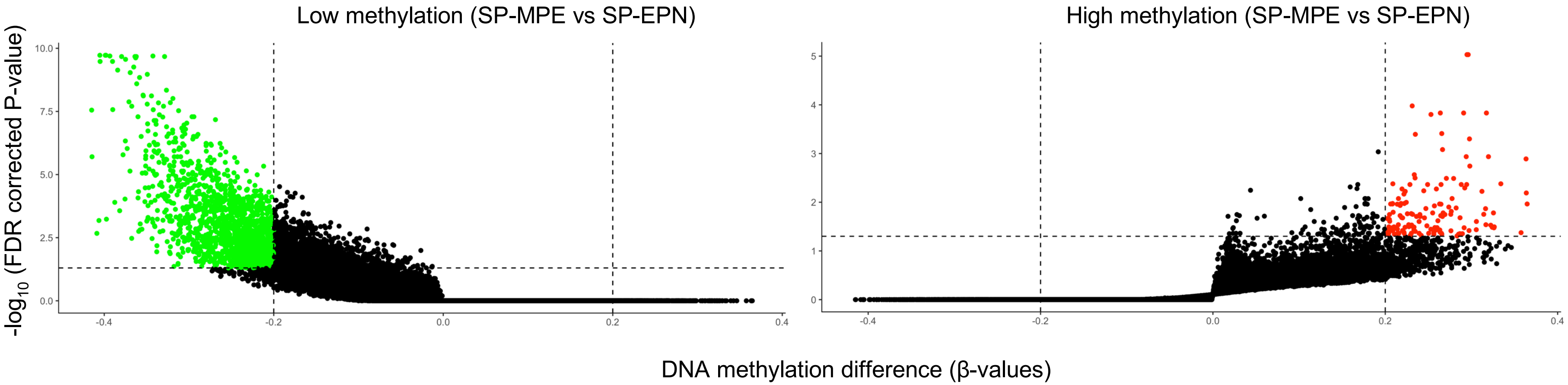

Supplementary figure S5. Differential methylated distal probes. Volcano plots showing DNA methylation differences (x-axis) and their statistical significance (y-axis) between SP-MPE and SP-EPN subgroups. Probes with low (left) and high (right) methylation levels in SP-MPE when compared to the SP-EPN subgroup are shown.

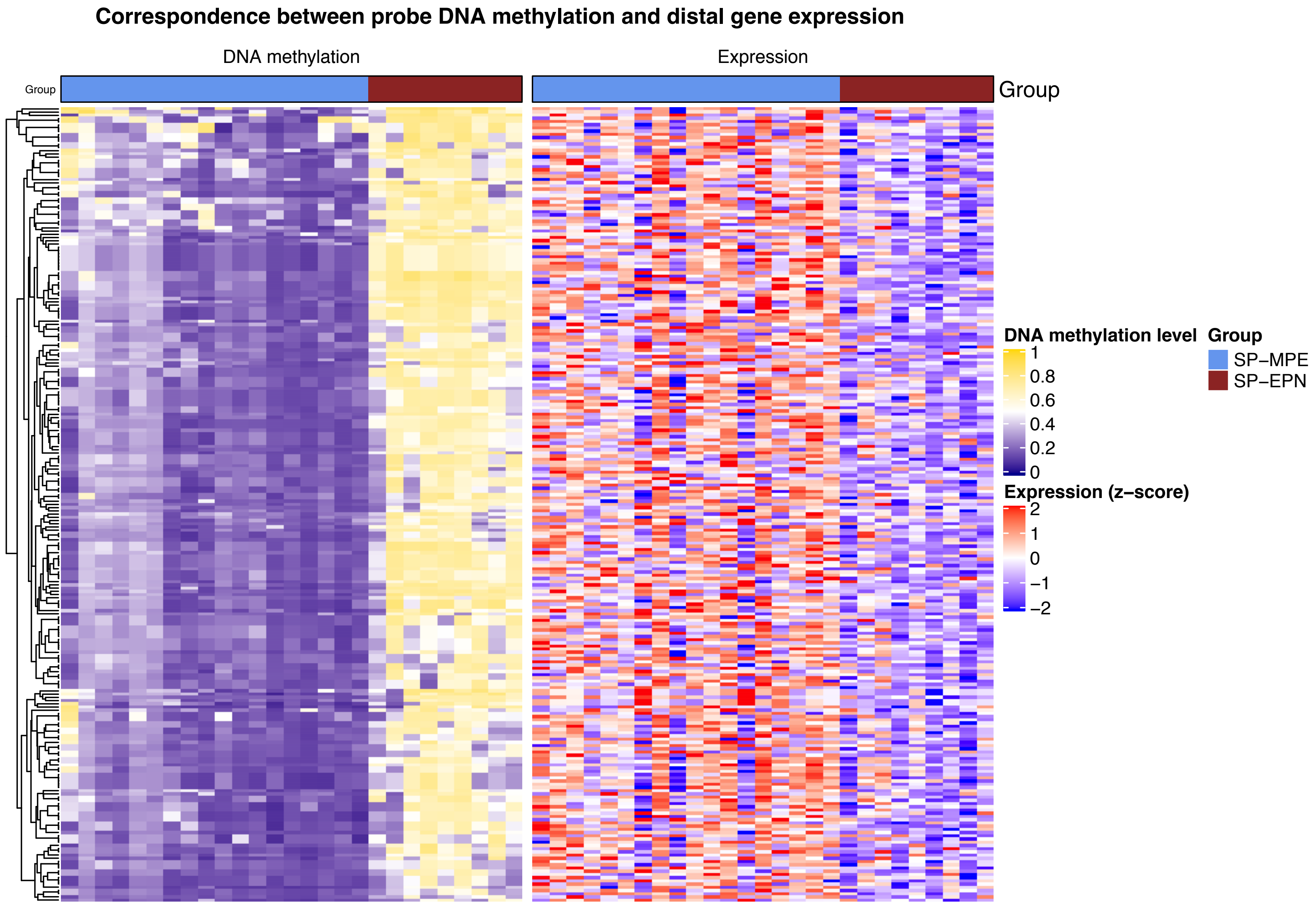

Supplementary figure S6. Probe-gene pairs showing inverse correlation between methylation and gene expression. Shown are the heatmaps for the significant DNA methylation probes (left) and gene expression (right) pairs at FDR < 0.05.
